# Multidecadal stability in tropical rain forest structure and dynamics across an old-growth landscape

**DOI:** 10.1371/journal.pone.0183819

**Published:** 2017-10-05

**Authors:** David B. Clark, Deborah A. Clark, Steven F. Oberbauer, James R. Kellner

**Affiliations:** 1 Department of Biology, University of Missouri-St. Louis, St. Louis, Missouri, United States of America; 2 Department of Biological Sciences, Florida International University, Miami, Florida, United States of America; 3 Department of Ecology and Evolutionary Biology, Brown University, Providence, Rhode Island, United States of America; Chinese Academy of Forestry, CHINA

## Abstract

Have tropical rain forest landscapes changed directionally through recent decades? To answer this question requires tracking forest structure and dynamics through time and across within-forest environmental heterogeneity. While the impacts of major environmental gradients in soil nutrients, climate and topography on lowland tropical rain forest (TRF) structure and function have been extensively analyzed, the effects of the shorter environmental gradients typical of mesoscale TRF landscapes remain poorly understood. To evaluate multi-decadal performance of an old-growth TRF at the La Selva Biological Station, Costa Rica, we established 18 0.5-ha annually-censused forest inventory plots in a stratified-random design across major landscape edaphic gradients. Over the 17-year study period, there were moderate differences in stand dynamics and structure across these gradients but no detectable difference in woody productivity. We found large effects on forest structure and dynamics from the mega-Niño event at the outset of the study, with subdecadal recovery and subsequent stabilization. To extend the timeline to >40 years, we combined our findings with those from earlier studies at this site. While there were annual to multiannual variations in the structure and dynamics, particularly in relation to local disturbances and the mega-Niño event, at the longer temporal scale and broader spatial scale this landscape was remarkably stable. This stability contrasts notably with a current hypothesis of increasing biomass and dynamics of TRF, which we term the Bigger and Faster Hypothesis (B&FH_o_). We consider possible reasons for the contradiction and conclude that it is currently not possible to independently assess the vast majority of previously published B&FH_o_ evidence due to restricted data access.

## Introduction

Have tropical rain forest landscapes been directionally changing through recent decades [1]? To answer this question for any lowland tropical rain forest (TRF) requires tracking its structure and dynamics through time. The within-forest heterogeneity in local environmental conditions that typifies TRF raises particular challenges. While the impacts of major environmental gradients in soil nutrients, climate and topography on tropical rain forest structure and function have been extensively analyzed, the effects of the much shorter environmental gradients typical of the mesoscale landscapes (1–100 km^2^ [2]) have received less attention. There are numerous studies looking at within-plot gradient effects [3–5], but there are many fewer studies that have systematically sampled mesoscale TRF landscapes [6–8].

Lowland tropical rain forests occur across a broad gradient of nutrient and topographic conditions [9]. Soils supporting TRF range from highly nutrient-rich to extremely nutrient poor, and the topography ranges from flat to steep and unstable. Sites on highly fertile soils have higher tree growth rates and stand turnover rates compared to sites on the most infertile soils [10,11]. What is much less studied and generally not quantified is the effect of the within-landscape gradients in nutrients and slope on tropical-forest structure and dynamics.

What degree of variance in soil nutrients and slope conditions is necessary to cause detectable changes in forest productivity and dynamics? Most tropical mesoscale landscapes contain a diversity of soil and topographic conditions [9]. For many reasons much of our understanding of these landscapes comes from a single small plot per local landscape [12]; such plots cover only a restricted range of landscape environmental conditions. If dynamics and productivity are sensitive to the within-landscape changes in soil nutrients and slope, single plots may give a biased picture of landscape conditions. Conversely, if dynamics and productivity are relatively insensitive to moderate variations in these conditions it becomes much easier to generalize specific results to larger spatial scales.

There has been surprisingly little focus on the effect of mesoscale landscape-scale variation in environmental conditions on TRF structure and function. Many analyses jump from the single plot to the aggregated global scale without knowledge of the accuracy of single plots as adequate samples of landscapes. There has been considerable controversy over using small plots to characterize the behavior of entire TRF landscapes [13–15], but to date there are few studies explicitly designed to measure TRF structure and dynamics at the landscape scale and through multiple decades.

We report here results from a study to characterize the behavior of a mesoscale old-growth TRF landscape at spatial scales ranging from <1 ha to ~ 500 ha and temporal scales from years to several decades across a moderate gradient of soil and slopes conditions. The basic experimental design of this work was a network of permanent forest inventory plots sited across gradients of soil nutrients and slope using a rigorous stratified random sampling protocol. The database we report on represents the most extensive long-term record to date of structure and annual dynamics of an old-growth tropical rain forest landscape (6 0.5 ha plots in each of 3 edaphic conditions, 18 consecutive annual censuses).

The landscape studied here was old-growth TRF in the Atlantic lowlands of Costa Rica at the La Selva Biological Station. Earlier work on forest productivity and dynamics at La Selva [16–18] predated our study by more than two decades. We used published results from these studies to extend the period of evaluation of this landscape to over four decades. During these four decades this forest experienced the extreme 1997–1998 El Niño event. We were thus able to study the forest's landscape-scale response to a significant climatic disturbance and could evaluate the magnitude of its effect on multi-decadal forest structure and dynamics.

In this aspect of the research we were interested in temporal trajectories of landscape structure and function over the last four decades. We were particularly interested in how trends in the structure and dynamics of this old-growth TRF landscape, arguably the most spatially and temporally sampled in the lowland tropics, compared with those from other TRF around the world in the same time period. Over the last four decades numerous studies have suggested increasing rates of biomass accumulation, mortality, recruitment and turnover in old-growth TRF around the tropics [1,14,19]. We term this paradigm the "Bigger and Faster Hypothesis" (B&FH_o_). Although based largely on meta-analyses of scattered small plots, the B&FH_o_ is inherently a hypothesis about the behavior of entire landscapes. There are many reasons to expect changes in the structure and dynamics of any given TRF landscape, including regional deforestation and selective timber extraction, defaunation due to hunting, past disturbance history, impacts of invasive species and changing regional patterns of temperature and precipitation. The B&FH_o_ however is concerned with patterns traceable to global, not local factors, and hence invokes changing global climate and atmosphere, particularly increasing levels of atmospheric CO_2_ concentrations, as possible drivers causing the reported directional trends in increasing forest biomass and dynamics [1,19]. We discuss the results from this research in relation to the B&FH_o_, and conclude that in general it is currently not possible to rigorously assess this paradigm due to the lack of publicly-accessible data and metadata.

## Materials and methods

Permits to conduct field work were granted by the Ministerio del Ambiente y Energía de Costa Rica (most recently permit 710-686-341). The research was carried out over an upland old-growth TRF landscape at the Organization for Tropical Studies' (OTS) La Selva Biological Station, Costa Rica. The forest is classified as Tropical Wet Forest in the Holdridge Life Zone system [20]. The long-term records of local meteorology at La Selva are available at (http://www.ots.ac.cr/). Annual rainfall over the last 52 years has averaged 4334 mm with no significant temporal trend. Annual daily mean temperature from 1993–2014 averaged 25.03°C and has increased significantly over this period at a rate of 0.13°C per decade (P_1-tail_ < 0.05).

The study area is considered old-growth forest (sensu [21]) and has been strictly protected for more than 50 years. As in many neotropical landscapes, pre-Columbian human settlements near the major rivers have been well documented at La Selva [22], and later settlements along rivers on the more fertile soils were common. Both types of settlers could have affected forest structure by hunting and by selective extraction of particularly useful species for food and timber [23]. All plots in this study were located on the poorer soils away from the more fertile recent alluvial floodplain.

In 1997 we established a network of 18 0.5-ha (50 m x 100 m) permanent forest inventory plots (the CARBONO plots), randomly stratified across the principal upland (non-swamp) gradients of slope and soil nutrients in this landscape (S1 Fig). Using a design based on strictly random coordinates, we sited plots within three landscape units: relatively flat areas on old alluvial soils; relatively flat areas on ridgetops on the more nutrient-poor residual soils; and on steeply-sloped sites on the residual soils. The plots' soil chemistry is discussed in detail in [24] (see their Appendix L for original soil chemistry data). In general soil nutrient stocks (P, K, Ca) are higher on the old alluvial soils compared to residual soils, and increase downslope from ridgetops to swales on the residual soils [24]. While there are significant differences in soil nutrient concentrations among the three edaphic categories, the gradient from richest to poorest stocks is only 2-3-fold for most nutrients (e.g., phosphorus, potassium). In contrast, slope conditions vary considerably among the landscape units, averaging <3° in the relatively flat plots and ~21° in the slope plots (plot-level slope data are on the CARBONO Project web page on the Organization for Tropical Studies website (http://www.ots.ac.cr/)).

In each plot all stems ≥10 cm diameter were identified, mapped, and measured annually from 1997–2014 (the study is ongoing). Annual mortality and recruitment was calculated following [25], and turnover calculated as the average of mortality and recruitment. In previous work we compared two methods of calculating Estimated Above Ground Biomass (EAGB), one based only on diameter [26] and one also incorporating wood density [27]; the estimates differed by only 8% [28]. The accuracy of either method is unknowable without extensive on-site harvest [29], so here we chose to base EAGB on the simpler Brown [26] allometry. All data analyzed here were originally collected by the co-authors' research projects. Further details of plot establishment and measurement protocols are available on the CARBONO Project web page. All data necessary to repeat the CARBONO plot analyses are in S1 and S2 Tables.

We analyzed interannual changes in EAGB at the plot level by calculating EAGB loss from mortality, EAGB added by new recruits, and EAGB biomass added or lost between annual censuses by the surviving individuals (growth). We emphasize that this analysis only addresses changes in EAGB stocks between censuses and does not in any way consider when this biomass was originally added to the forest system. For example, recruits entered this study when they reached ≥10 cm trunk diameter, but the <10 cm diameter portion of their biomass was formed years or decades earlier. Similarly, most dying individuals were present when the study began, and most of their biomass was formed decades or centuries prior to this study.

We placed the forest dynamics over the 17 years of our study (1997–2014) in a longer temporal context by relating our findings to those from earlier studies of forest dynamics at La Selva [16–18]. In 1969 a 4.4-ha plot was established on flat old alluvial soils (OTS Plot 1, S1 Fig) and a 4.0-ha plot was sited on an area of ridgetop and slope topography on residual soils (OTS Plot 3 [16]). These plots were subsequently recensused in the 1980s [16–18]. We compared data from the 6 CARBONO plots on old alluvial soil with the 4.4-ha plot on old alluvial soils, and compared the data from the 12 CARBONO plots on ridgetops and slopes on residual soils with the data from the 4.0-ha plot on residual soils.

## Results

### Intra-landscape variation in forest structure and dynamics

The dynamics and physical structure of the CARBONO plots varied significantly between the two soil types (residual, old alluvium), but did not differ in any aspect between the plateau- and slope-plots on residual soils (Table 1). The plots on old alluvial soils had fewer and larger trees than plots on residual soils and were less dynamic, with lower rates of recruitment, mortality, and turnover of individuals, and longer residence times of biomass and basal area. In spite of the numerous differences in structure and dynamics between plots on residual and alluvial soil, there were no significant differences in woody productivity (cf annual rates of diameter growth and basal area and estimated biomass addition, Table 1) among the three edaphic categories.

**Table 1:** Forest structure and dynamics by soil type and topography.

Variable	Flat alluvial mean	SEM	Ridgetops mean	SEM	Slopes mean	SEM	Soil effect	Slope effect
Stem Number	201.4	2.3	262.1	1.8	272.8	2.0	***	NS
Mean stem diameter mm	230.2	1.5	198.8	0.9	203.9	0.9	**	NS
Basal area (m^2^)	12.3	0.1	11.1	0.1	12.1	0.1	NS	NS
EAGB Mg	88.1	0.9	74.3	1.0	80.8	1.2	*	NS
Mean annual diameter growth rate mm	2.9	0.0	3.1	0.1	3.0	0.1	NS	NS
Mean annual BA addition/plot m^2^	0.266	0.003	0.286	0.005	0.304	0.005	NS	NS
Mean annual EAGB addition per plot MG	2.11	0.03	2.14	0.04	2.31	0.04	NS	NS
Mean annual mortality %	2.10	0.13	2.56	0.23	2.40	0.14	*	NS
Mean annual recruitment %	1.87	0.09	3.11	0.17	2.35	0.13	*	NS
Mean annual turnover %	1.98	0.08	2.83	0.16	2.37	0.09	*	NS
Basal area residence time (yrs)	41.0	0.6	32.8	0.8	34.1	0.9	*	NS
EAGB residence time (yrs)	39.9	0.6	32.6	0.8	33.3	0.9	*	NS

SEM = standard error of the mean, EAGB = Estimated above-ground biomass, BA = basal area. All data are from 18 consecutive annual censuses, 6 0.5 ha plots per edaphic category. Edaphic category statistics are based on analysis of the mean values of each variable per 0.50 ha plot. Repeated measures ANOVA were based on annual plot-level values with plots as subjects and edaphic category as the treatment. Soil effect refers to tests for difference between plots on flat topography on the two soils (alluvial vs. ridgetop plots); slope effect refers to tests for differences between plots on similar soils and contrasting flat versus steeply-sloped topography. Residence times are calculated as each year's basal area or EAGB divided by that year's plot-level basal area or EAGB increment. Residence times apply to EAGB and basal area in the ≥10 cm diameter portion of each individual.

* P<0.05,

** P<0.01,

*** P<0.001.

**Fig 1:**
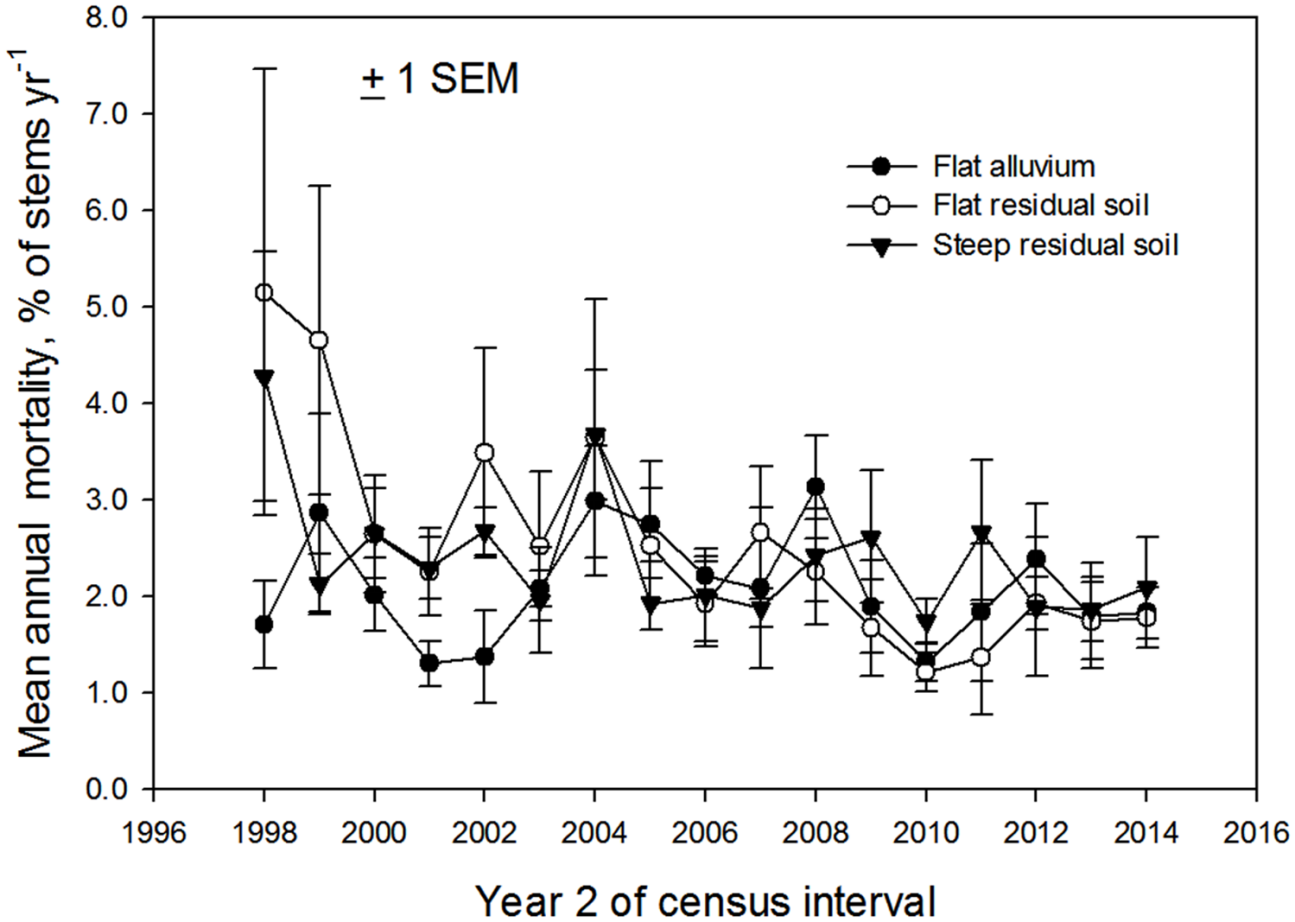
Mortality rates by edaphic category. Rates of annual tree mortality (% of stems) in the three landscape units, 1997–2014 (N = 6 0.5 ha plots in each).

The lack of intra-habitat difference in productivity suggests that the intra-habitat differences in forest dynamics were principally due to differences in local rates of disturbance. It is not clear how soil type per se could influence gap-formation rates, although it is possible that soil moisture stress is higher on ridgetops than old alluvial terraces [24] and that this promotes higher tree die-off in periods of greater water limitation. Higher mortality of large trees on ridgetops during droughts has been reported from an Asian forest [30,31]. An alternative explanation for higher mortality in the ridgetop plots is the geographic location of those plots compared to the old alluvial plots (S1 Fig). The ridgetop plots are at higher elevation and could be more exposed to wind and/or lightning than either the old-alluvial or slope plots. The largest mortality events during the study period occurred during the first half of the study and were concentrated in ridgetop plots (Fig 1).

### Interannual variation in forest structure

**Fig 2:**
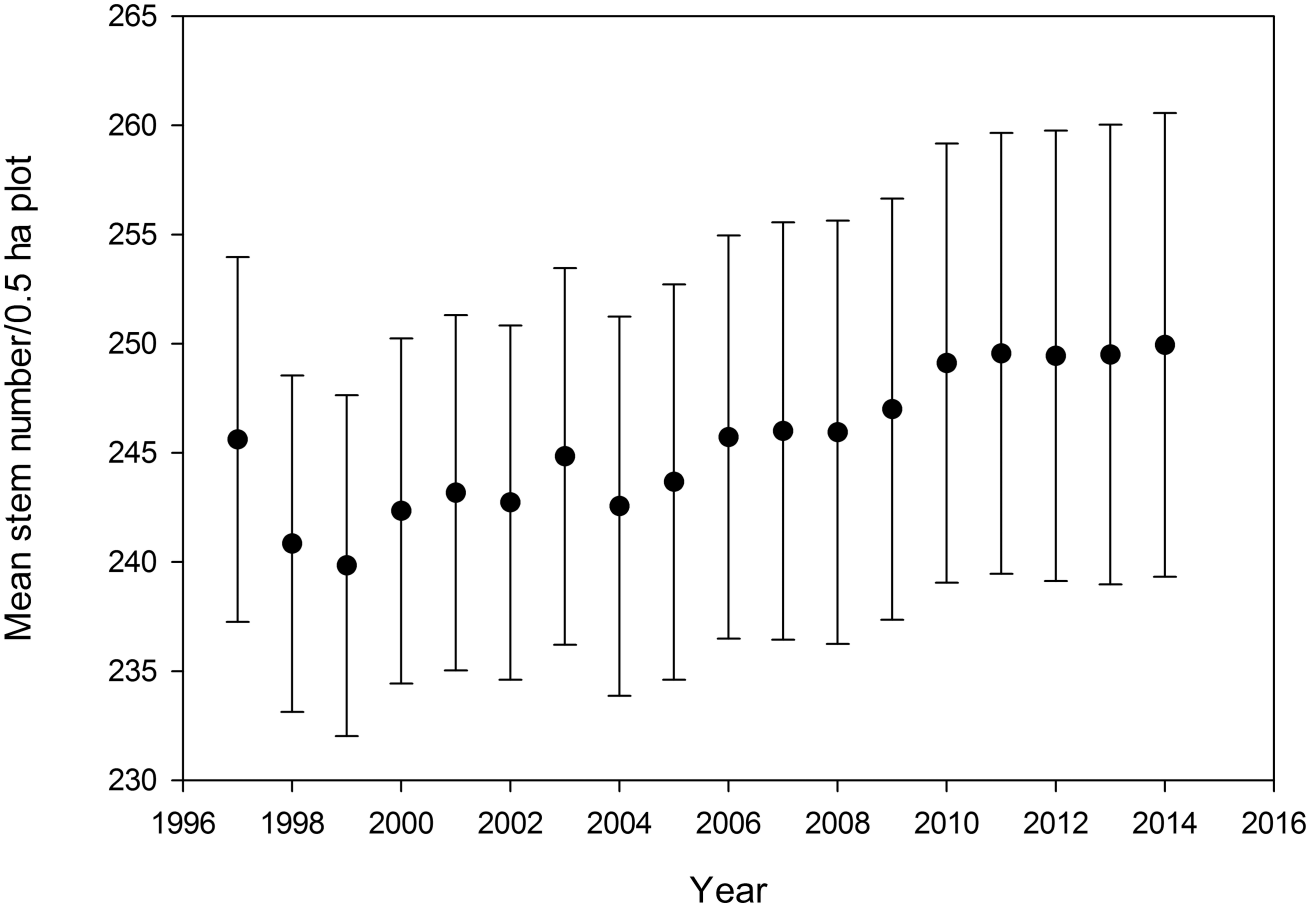
Landscape-scale stem density through time. Mean number of stems ≥10 cm diameter in 18 0.5-ha plots ± 1 SEM.

Most aspects of forest physical structure and demographic processes responded to the 1997–1998 strong Niño event. There was a 2% decrease in stem number in the two years following the 97–98 Niño (Fig 2). After this decrease, stem density increased gradually over the following ten years to ~ 1.8% above the initial density. Stem density was stable for the last four years of the study. The landscape-scale increase in stem number was driven completely by the ridgetop plots; the plots on slopes and old alluvium showed slight decreases in stem number (S2 Fig).

**Fig 3:**
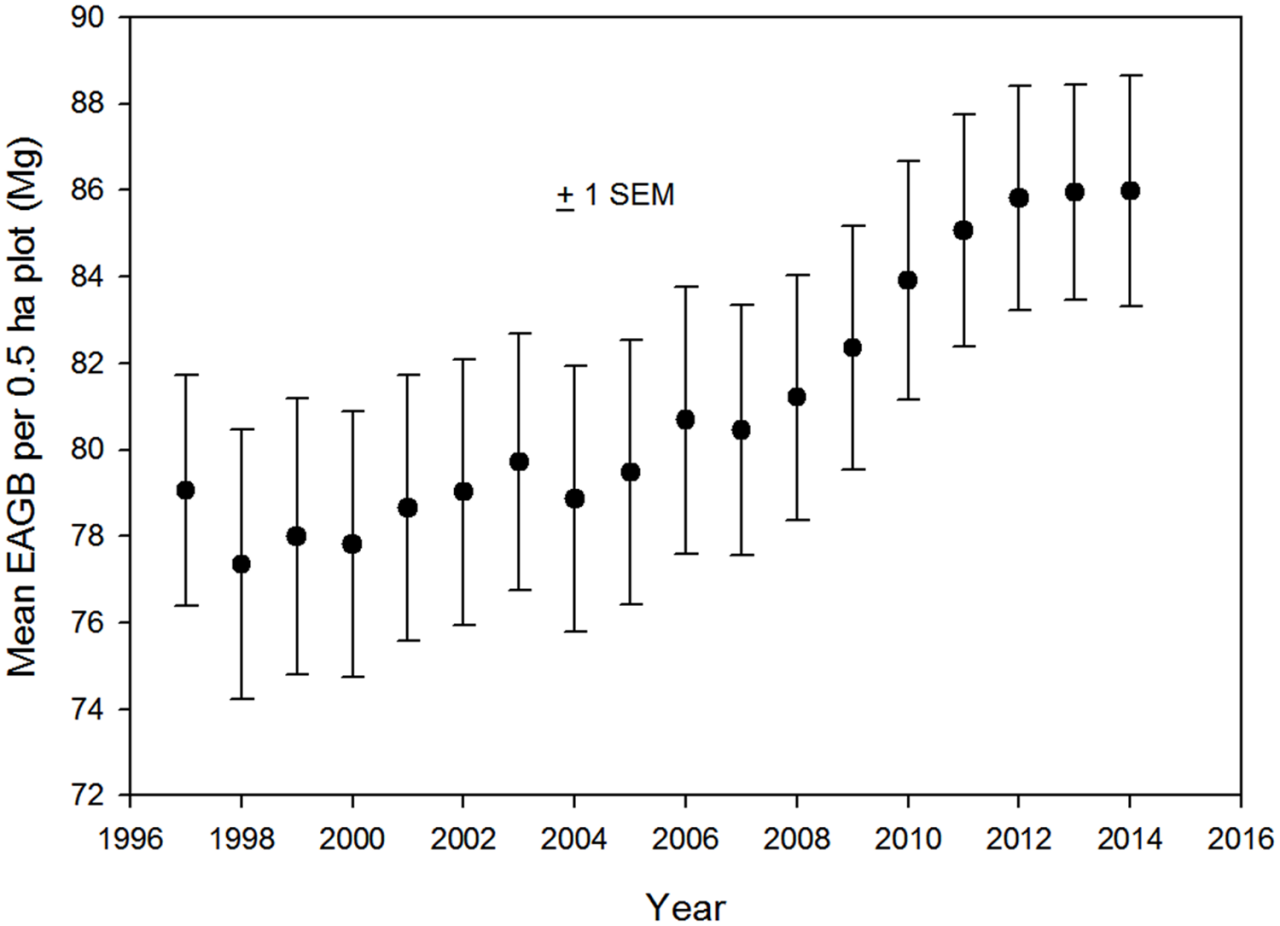
Landscape-scale estimated above-ground biomass through time. Mean Estimated Above-Ground Biomass ±1 SEM in 18 0.5-ha plots in old growth at the La Selva Biological Station, Costa Rica.

Dynamics of basal area and estimated above-ground biomass (EAGB) followed a pattern similar to stem density, i.e. a small decrease following the 97–98 strong Niño, a slow increase for the next decade followed by a plateauing in the last three years of the study (Fig 3). Both basal area and EAGB increased to approximately 8–9% above their initial values. Unlike stem density, the increase in basal area and EAGB was similar in plots on all three edaphic conditions (S3 Fig).

### Interannual variation in forest dynamics

**Fig 4:**
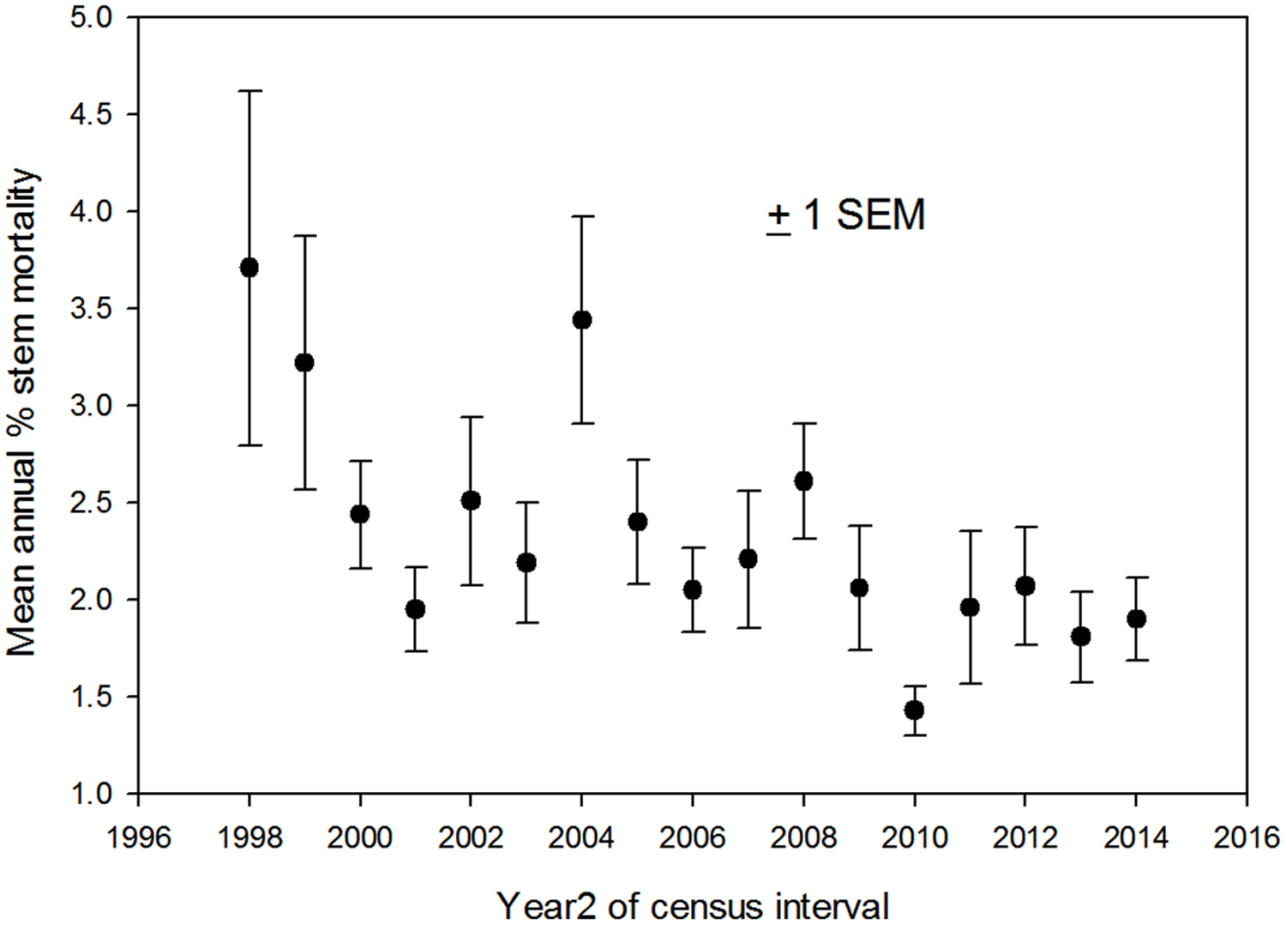
Landscape-scale mortality patterns. Mean rates of mortality ± 1 SEM in 18 0.5-ha plots in old-growth forest at the La Selva Biological Station, Costa Rica, from 1997 to 2014.

At the landscape scale mortality was highest in the 1997–1998 Mega-Niño, spiked again in 2004, and then remained low through the following ten years (Fig 4). There is no suggestion of increasing mortality through time. Mortality was relatively constant through the 17 years in the plots on old alluvium and slopes, while it decreased strongly over time in the ridgetop plots (Fig 1). Rates of recruitment and of turnover (the mean of mortality and recruitment) both increased after the Mega-Niño and then trended downward for the last decade (S4 and S5 Figs)

**Fig 5:**
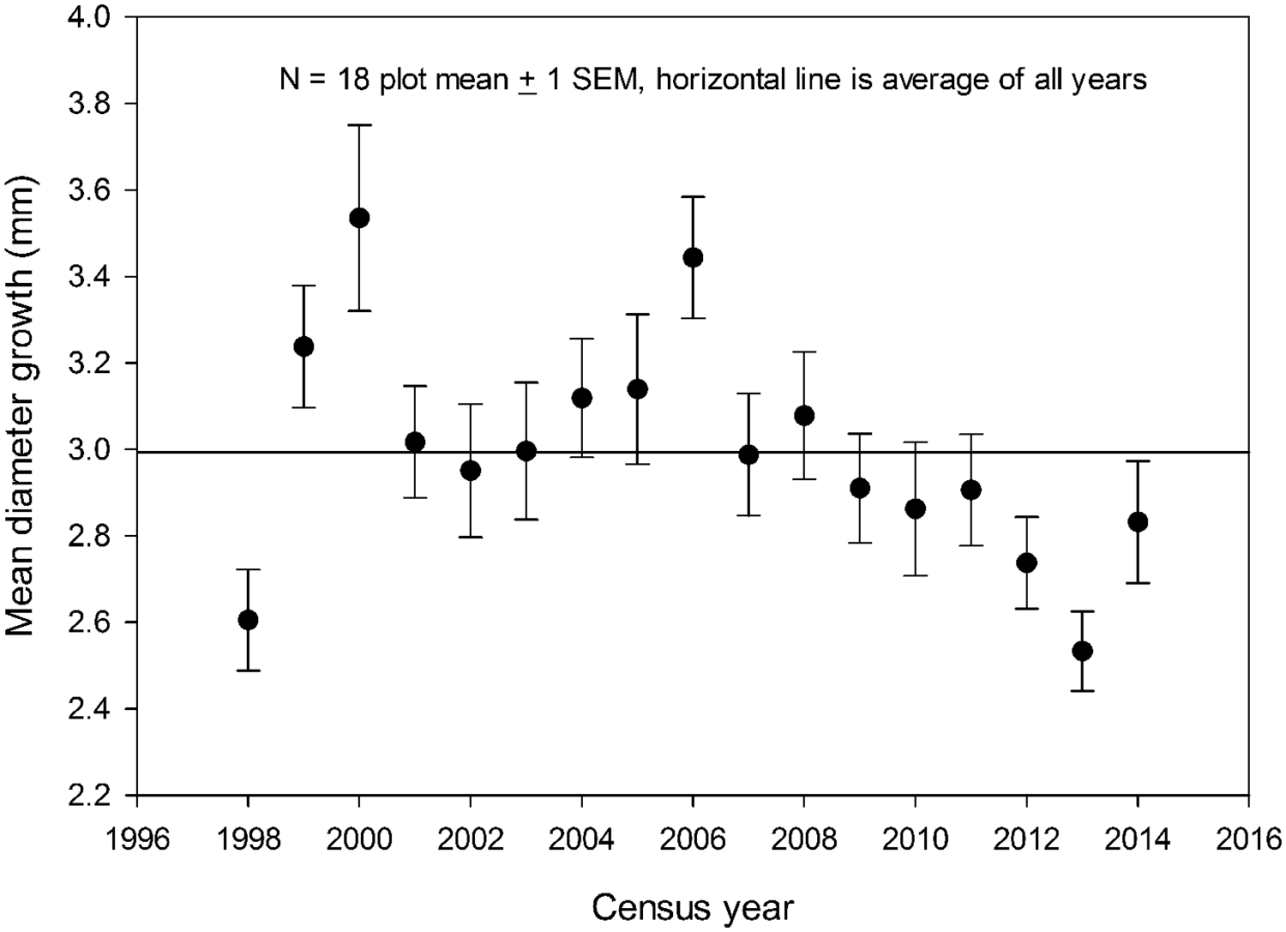
Landscape-scale diameter growth rates. Mean rates of stem diameter growth (mm) ± 1 SEM for all woody stems ≥10 cm diameter in 18 0.5-ha plots in old-growth forest at the La Selva Biological Station, Costa Rica, from 1997–2014. The horizontal line indicates the 17-yr mean of the annual diameter growth rates.

Diameter growth rates increased after the major mortality events in 1997–98 and 2003–04 (Fig 5). Otherwise there was no systematic trend through time, with growth rates tending downwards in the latter years.

**Fig 6:**
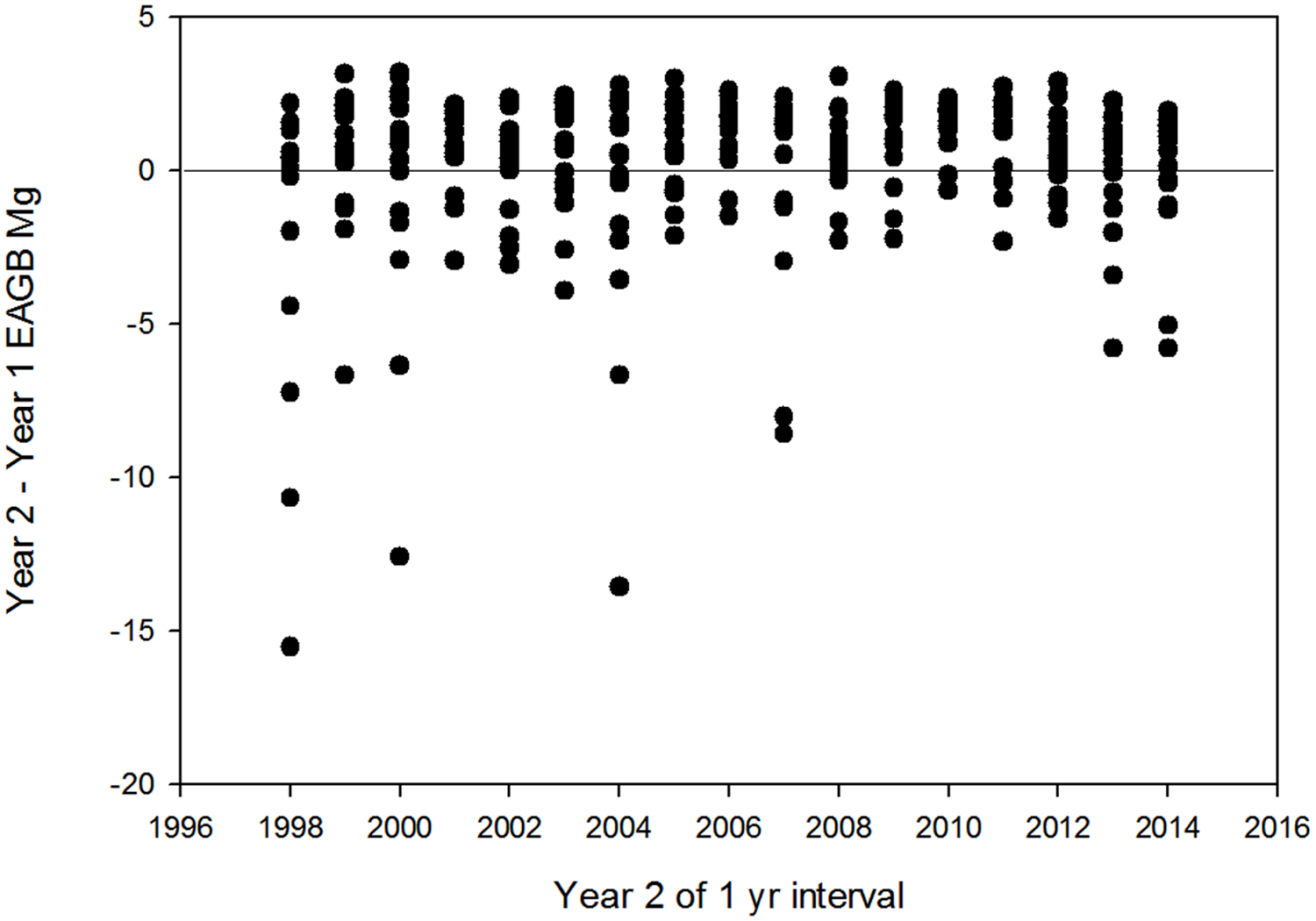
Interannual changes in plot EAGB. Annual changes in plot EAGB (Mg per plot) in each of the 18 0.5-ha forest inventory plots in old-growth forest at the La Selva Biological Station, Costa Rica.

We analyzed the factors associated with interannual changes in EAGB at the plot level by considering first the magnitude of all changes, and then the relative impact of sources of interannual changes in EAGB. Over 70% of the plot-level inter-year changes in EAGB were positive (Fig 6). Interannual plot-level EAGB losses averaged ~70% larger than plot level EAGB increases, and maximum losses were five times larger than maximum biomass increases.

**Fig 7:**
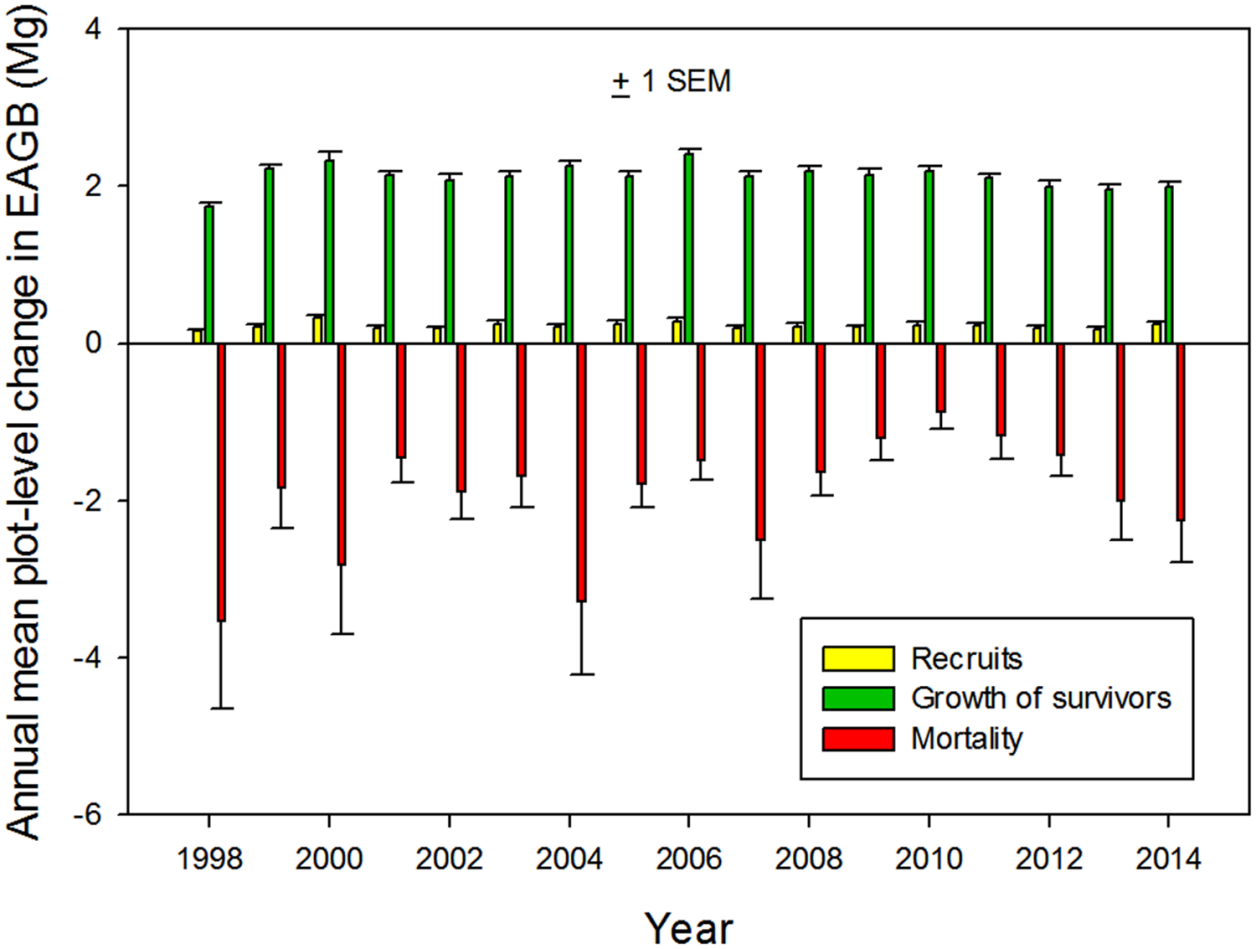
The three components of the landscape-scale mean annual changes in plot EAGB. Interannual dynamics of biomass change in 18 0.50 ha plots across an upland old-growth landscape at the La Selva Biological Station, Costa Rica from 1997–2014. Biomass loss by mortality and addition by recruits are calculated based on the entire EAGB of the involved individuals, while growth of survivors is the summed calculated increase in EAGB of the stems that survived that census interval.

To determine the relative importance of factors causing interannual changes in plot biomass we analyzed changes in EAGB due to recruitment, mortality and biomass addition by survivors. Loss of biomass by mortality and biomass addition through growth of survivors were the principal factors associated with interannual changes in plot EAGB (Fig 7). Mortality loss of EAGB averaged 3.87 Mg ha^-1^ yr^-1^ per 0.5-ha plot while addition of EAGB by growth of survivors averaged 4.25 Mg ha^-1^ yr^-1^ and recruits added 0.44 Mg ha^-1^ yr^-1^. EAGB loss by mortality was more than five times more variable than EAGB addition by growth of survivors (CV 38 vs. 7). The interannual changes in plot-level EAGB were thus principally driven by mortality. In spite of the known sensitivity of stem growth to small climatic variations at La Selva [28,32], addition of biomass by survivors was remarkably constant across the landscape, both in a given year (see error bars on Fig 7) and among the 17 years (CV of annual means = 7).

### Forest change on multi-decadal time scales

To understand landscape-scale forest dynamics in a longer-term context, we compared the results from 17 years of detailed annual measurements of forest structure and process with previously published data from this site. We also compared the long-term patterns observed over this landscape with those observed over other tropical landscapes over a similar time period [1,14].

**Fig 8:**
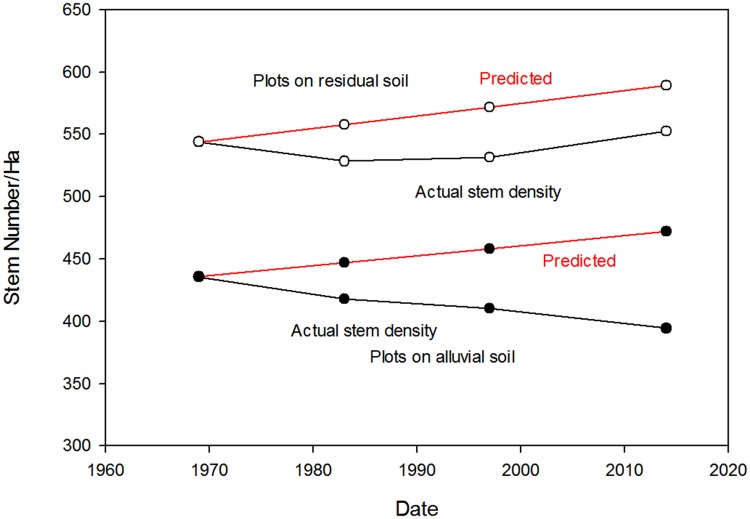
Historical trends in stem density. Historical trends in stem density (number per hectare). Stem density in old-growth forest over more than four decades at the La Selva Biological Station, Costa Rica. Data for 1969 and 1983 for the alluvial soils and for the residual soils are from OTS Plots 1 and 3, respectively [16]. The predicted increase in stem density by the B&FH_o_ was 0.18% linear increase per year [14].

Stem density increased 1.6% over the 45-yr interval for plots on residual soils, while it declined 9.5% over the same period for plots on alluvial soils (Fig 8). The small increase on residual soils was much less than changes predicted from observations of Amazonian forests over a similar time period (Fig 8), and the changes on the alluvial soil were in the opposite direction of the B&FH_o_.

**Fig 9:**
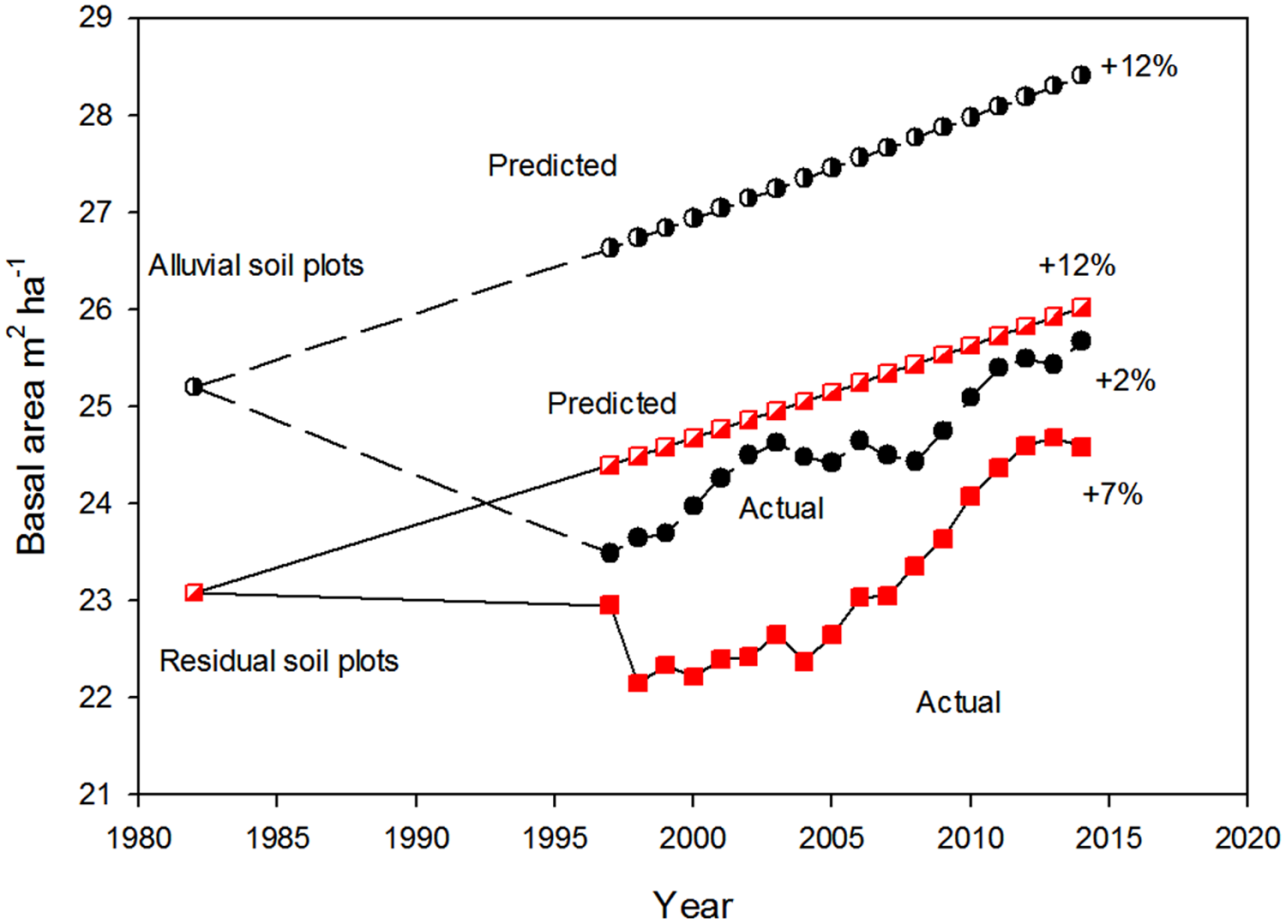
Inferred historical trends in basal area. Long-term change in basal area over an old-growth tropical rain forest landscape at the La Selva Biological Station, Costa Rica. Data for forest on alluvial soil in1982 are from a 4.4 ha plot (OTS Plot 1 [18]), subsequent data from the 6 0.5 ha CARBONO project alluvial plots. Residual soil plots are OTS Plot 3 (4.0 ha, [18]) in 1982 and subsequently the 12 CARBONO project 0.5 ha ridgetop and slope plots. Predicted values are based on long-term trends reported from forest plots in Amazonia [33].

Based on this composite analysis, over a 32-year interval, basal area in the La Selva old-growth forest increased 2.0% on the old alluvial soil and 6.6% on the residual soil (Fig 9). Both increases are much lower than those inferred from plots in Amazonian forests (0.38% year^-1^ linear increase [33]). In addition, at La Selva the inferred increase in basal area was much less on the more fertile old alluvial soils, opposite to the expectation of a greater response to increasing atmospheric CO_2_ on more fertile soils [34].

**Fig 10:**
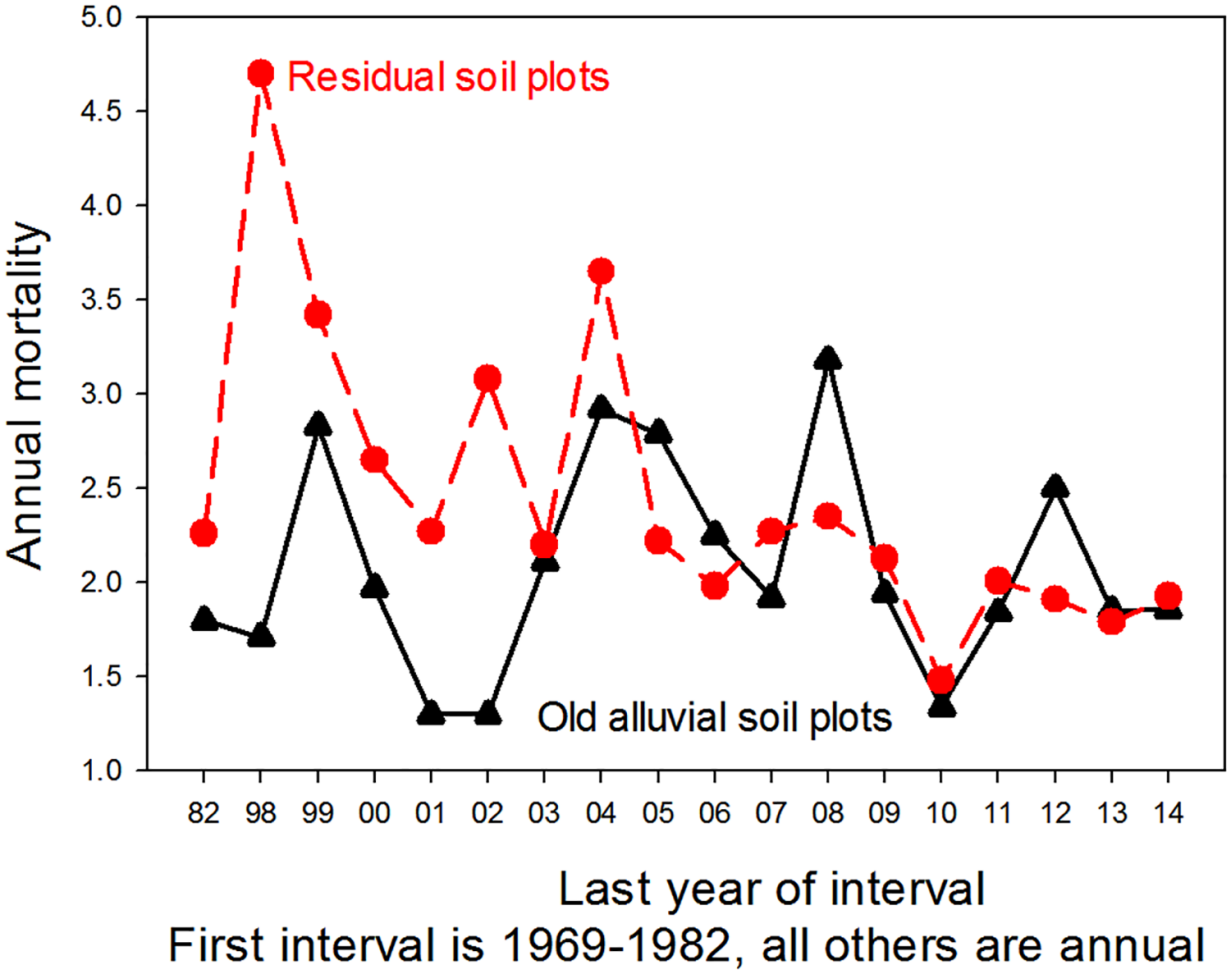
Multidecadal trends in mortality. Multidecadal trends in rates of % annual tree mortality in old-growth forests on residual and alluvial soils at the La Selva Biological Station, Costa Rica. Data from 1969–1982 are from OTS Plot 1 (4.4 ha, alluvial soil) and OTS Plot 3 (4.0 ha, residual soil [17]). Data from 1997 onward are from the 6 0.5 ha CARBONO Project plots on alluvial soil and the 12 0.5 ha plots on residual soil (ridgetops and slopes). Old alluvial data are in black, residual soil data in red.

Using the historical data we compared the stem death rates from three and four decades ago to those observed over the last two decades. Over the 45-yr period there was substantial variation in forest mortality rates on both alluvial and residual soils (Fig 10). Contrary to reports from other tropical forests [1], there was no decadal increase in stem mortality on either the alluvial or the residual soils. Patterns in recruitment were similar to those for mortality, including the lack of a long-term directional trend (S6 Fig).

**Fig 11:**
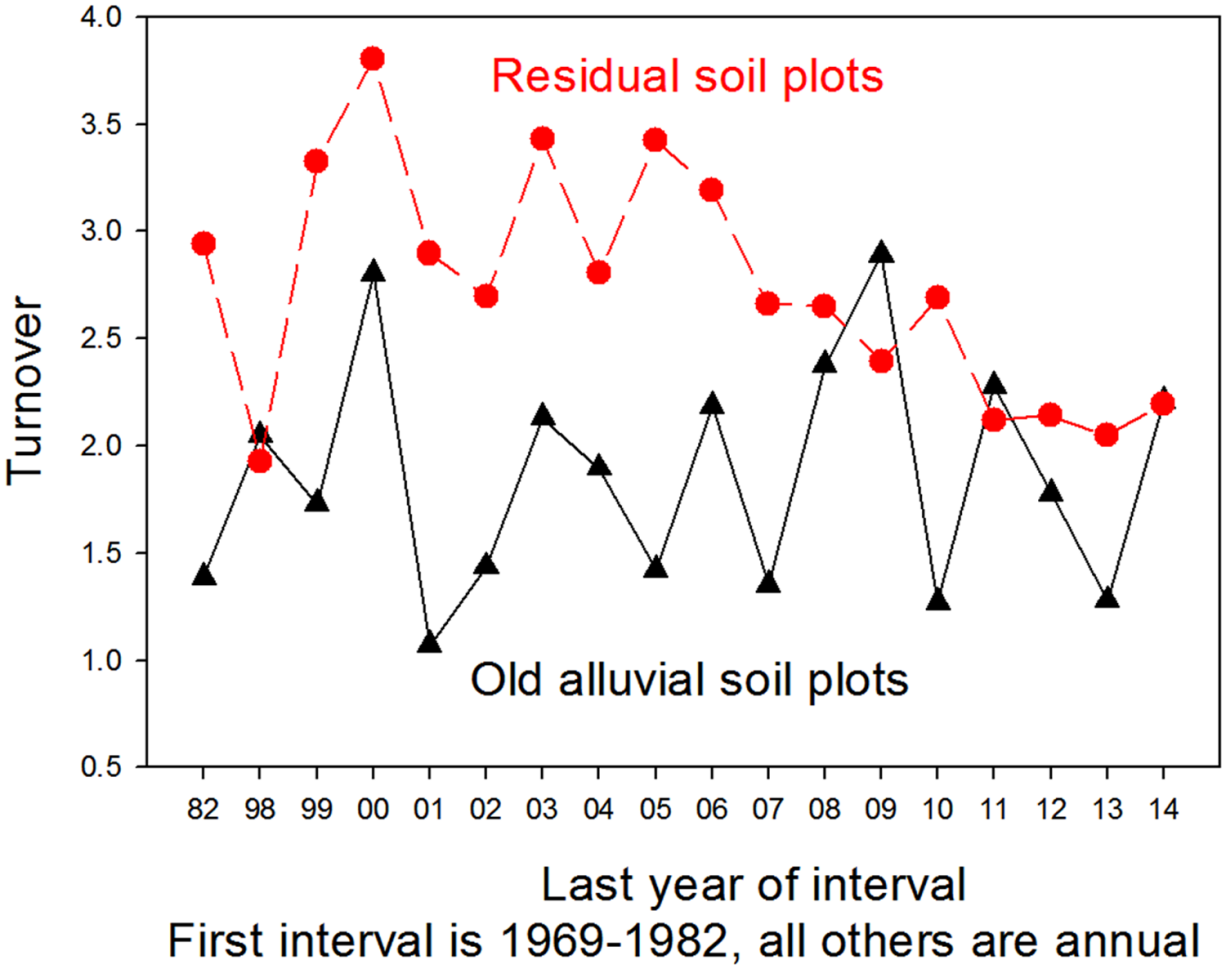
Historical trends in forest turnover. Temporal changes in annual forest turnover rates over an old-growth tropical rain forest landscape at the La Selva Biological Station, Costa Rica. Data for forest on alluvial soil from 1969–1982 are from a 4.4 ha plot (OTS Plot 1 [17]), subsequent data from the 6 0.5 ha CARBONO project alluvial plots. Residual soil plots are OTS Plot 3 (4.0 ha [17]) from1969-1982 and subsequently the 12 CARBONO project 0.5 ha ridgetop and slope plots. Residual soil data are in red, alluvial soil data in black.

There was a substantial difference between soil types in the long-term dynamics of stem numbers across the La Selva old-growth landscape. The forest on residual soils had a higher long-term mean rates of mortality than plots on alluvial soils (2.46% per year ± 0.19 SEM vs. 2.08% per year ± 0.13; N = 19 intervals) and of recruitment (2.7% per year ± 0.13 vs. 1.84% ± 0.13). As a result, over the last 45 years turnover on the residual soils averaged almost 50% higher than on alluvial soils (2.74 ± 0.13 vs. 1.86 ± 0.13). Although there was substantial variation among years and between soil types, there was no suggestion of a multi-decadal increase in turnover on this landscape on either soil type (Fig 11).

## Discussion

### Intra-landscape variation in upland forest physical structure and function

The CARBONO project was explicitly designed to measure the effects of contrasting soil and slope conditions on forest performance across the La Selva old-growth landscape. The within-forest soil nutrient gradient was ≤ two-fold for most nutrient stocks [24], while slope conditions contrasted on average by ~18*. Over the 17-year period covered by this study there was no significant difference in above-ground woody productivity across these gradients (Table 1). If there were differences among these edaphic categories in terms of effects on woody productivity, they were not detectable with the degree of site and temporal replication reported here. At this point we conclude that the 2–3 fold nutrient gradients across the treatments were not sufficient to cause observable effects on above-ground woody production.

In contrast, there were significant soil-related differences in local forest structure and dynamics (Table 1). Stem turnover was 31% faster on the residual soil plots; these plots had 33% more stems and a mean stem diameter 13% smaller than on the alluvial-soil plots. Faster dynamics on the residual-soil ridgetops and slopes at La Selva were also found by Silva et al. [35] in analyses of annual canopy heights over a 12-year period (1999–2010) within our study interval. Considered together, these findings point to local disturbance over the last several decades as a key proximate factor determining the current-day physical structure and dynamics of this old-growth landscape.

This study provides error-bounded estimates of the magnitude of temporal and spatial variation in structure and function within a relatively homogeneous upland old-growth TRF landscape. The intra-landscape differences in structure and function demonstrated here show the magnitude of error that would be caused by generalizing from a single plot in one habitat type to the entire landscape. For example, over the 17-year CARBONO study period, on the alluvial soils stem number decreased through the period, turnover averaged 1.98%, tree diameters averaged 230 mm, and mean stem number per 0.5-ha plot averaged 201. In contrast, in the plots on residual-soil ridgetops, mean stem number increased through time, and mean stem diameter was 14% lower and turnover and stem density were 43% and 30% higher respectively than in the alluvial-soil plots (Table 1 and S2 Fig). Whether or not this magnitude of difference in intra-landscape structure and function is significant will depend on the level of accuracy a given study seeks to achieve. For global comparisons across a very large range of environmental conditions such variance may be minor importance. On the other hand, attempts to compare similar TRF landscapes clearly need to take into account the potential for intra-landscape variations of the order of magnitude found in this study.

### Annual, decadal, and multidecadal variance in upland forest physical structure and function

This study was based on intensive monitoring of a mesoscale tropical rain forest landscape, with 20 censuses spanning 45 years (Fig 10). Due to the number of replicate plots, their siting across an edaphic gradient and the long series of repeat censuses, we can draw several conclusions about the trajectory of this landscape.

Considered as a whole, over the last 45 years the La Selva old-growth forest has shown little long-term change in physical structure or demographic rates at the landscape level (Figs 8–11, S6 Fig). Rates of mortality, recruitment and turnover varied several fold through the decades, and there was no directional trend in these rates. Stem density was essentially unchanged on residual soils, while it declined on the old alluvial soils. Basal area increased 2–7% over the 45 year interval (Fig 9) and plateaued in the last years of the study period. Over the last 17 years diameter growth rates varied about 40% among years but with no suggestion of a directional trend. In summary, the combined data show a landscape with some degree of annual and decadal variance in structure and function but little noticeable change at the multidecadal time scale.

The largest differences in forest structure and dynamics occurred at the annual and sub-decadal time scales (cf Figs 5 and 10). The 1997–98 strong ENSO event clearly impacted both the forest structure and tree demographic rates. The effect of this extreme climatic event on forest biomass was large: a decrease of 3.43 Mg EAGB/ha between 1997 and 1998 (Fig 3 scaled to 1 ha). In contrast, Phillips et al [19] found that Amazonian forests "actually impacted" by an intense Amazonian drought in 2005 lost an average of 1.62 Mg EAGB/ha, or less than half the landscape-level impact of the strong Niño at La Selva.

Nevertheless, the effects of the strong ENSO over the La Selva old-growth landscape lasted less than a decade in attributes of physical structure (cf Fig 3) and only a few years for demographic rates and woody production (cf Figs 4 and 5). Three studies using completely different data types have reached similar conclusions about the stability and resilience of this landscape in recent years. Kellner et al. [36] used repeat lidar data from both 1997 and 2006 and found that the canopy-gap size-frequency distribution was unchanged and close to equilibrium over that interval. Over a shorter time period (1998–2005) and using repeat lidar data, Dubayah et al. [37] found this old-growth landscape was stable in terms of canopy height and EAGB. Silva et al. [35] analyzed ~ 48,000 annual ground-based estimates of canopy height from 1999 to 2010. They found a departure from canopy height equilibrium during 2–3 years after the 1997–98 ENSO event, followed by a return to equilibrium.

For sub-decadal intervals the largest factor influencing forest structure was the effects of local disturbances. Interannual changes in plot basal area and EAGB are primarily driven by mortality (Figs 6 and 7). Our findings from the La Selva old growth are therefore consistent with Körner's [13] "Slow in, fast out" hypothesis. The importance of mortality in determining forest structure on subdecadal time scales has also been found in other TRF [38–39].

### Long-term performance of the La Selva landscape and the Bigger and Faster Hypothesis

The old-growth lowland tropical rain forest landscape at La Selva has been largely stable over the past 45 years in terms of physical structure and demographic rates. While there is substantial annual and multiannual variation in these forest attributes, particularly in relation to local disturbance, at the multidecadal scale we found almost no directional trends. This old-growth landscape thus largely does not conform to the predictions of the Bigger and Faster Hypothesis. Rates of recruitment, mortality, turnover and tree diameter growth did not increase over time. There were small increases in basal area and in stem number on the residual soil plots, but these are much less than reported from other tropical forests [14] and the alluvial soil plots showed decreasing stem number.

Why does the La Selva upland old-growth landscape largely not conform to the B&FH_o_? One possibility is that the La Selva landscape is in some way different from other tropical landscapes in ways that causes it to exhibit stability while other tropical landscapes are accumulating biomass and increasingly dynamic. We can think of no plausible factor that would cause such atypical stability. In general soils at La Selva are on the more nutrient rich side of the spectrum of soils underlying the forest plots used to the B&FH_o_ analyses [40], and if anything richer soils should accelerate responses to rising CO_2_ levels [34], which is a hypothesized driver of increased forest biomass and dynamism [1].

Another possibility is that the plots used in the meta-analyses for the B&FH_o_ analyses were not random samples of their landscapes. For example if those small, unreplicated plots were biased towards towards older successional areas like regenerating floodplains or particularly mature patches ("majestic forests"), the plots' behavior could differ from that of the larger landscape. If different habitat types across a landscape differ in structure or performance, a single plot cannot capture this variation. At our study site for example, a random plot established on ridgetops sites would likely have shown increasing stem number over the last two decades, a site on alluvial soils would have shown decreasing stem density, and a site on a slope would be expected to have shown virtually no change (S2 Fig).

Many of the plots used in the B&FH_o_ analyses were established decades ago. To our knowledge the metadata documenting the exact siting criteria and the protocols for the field implementation of those criteria are not publically available for any long-term tropical forest plot study except for the CARBONO Project (see CARBONO project website Plot Design and Survey Implementation). While it is thus impossible to objectively evaluate the protocols used to site nearly all long-term tropical-forest plots, we think it highly unlikely that most were sited using a rigorous random protocol at the local landscape scale. Since many of the older plots are unreplicated, it is also unknown how these single plots relate to the landscape-scale structure and dynamics in those forests.

It is possible to evaluate the current-day representativeness of plot sites in relation to the larger landscape using remotely-sensed data. Marvin et al. [15] compared structural metrics derived from large spatial scale lidar data with those from 1-ha forest inventory plots and found significant bias in the ground data. Their simulations suggested that between 10 and 100 1-ha plots would be necessary per landscape to overcome the small-plot sample bias. While their study has not yet been replicated in other tropical forest landscapes, it clearly suggests the potential for bias when a single small sample is considered to represent an entire landscape.

For plot-based studies, landscapes can be efficiently sampled with a random sampling design stratified across the major within-forest environmental gradients known or suspected to influence forest structure and dynamics. This approach however is very rare in tropical forests; the CARBONO study is so far exceptional in using a stratified random design with many annual resamplings over decadal time scales. An alternative approach is the establishment and monitoring of a single very large forest plot per landscape. In general plot bias should decrease as plot size increases [41], since an increasing proportion of the within-landscape variance is sampled. Even very large plots, however, cannot contain the total landscape variance. If a large plot includes significant areas of secondary or disturbed forests, conclusions from the plot about the behavior of old growth can be affected.

Two large plots with long and intensive re-measurement histories are the 50-ha plot on Barro Colorado Island, Panamá (BCI [42]), and the 50 ha Pasoh plot in Malaysia [43]. Over a 20-yr period, the change in biomass on BCI was not distinguishable from zero, while at Pasoh biomass was found to have increased over a 14-yr period [44]. The results from these large plots were thus inconclusive for the B&FH_o_.

Additional factors that could produce erroneous conclusions about trends in tropical-forest biomass and dynamics have been reviewed previously [45–47]. A definitive assessment of the relevant plot data will require additional studies like that of Marvin et al. [15] to evaluate how specific plots relate to their larger landscapes, as well as analyses of the field evidence that has been cited as supporting the B&FH_o_ (e.g., the plots' raw data, field data sheets, and protocols for the field measurements and site location protocols). To our knowledge, to date all of the relevant field data are publically available only for the CARBONO Project (see CARBONO website).

### The future of tropical forest science and assessing the Bigger and Faster Hypothesis

The La Selva upland old-growth landscape was largely stable in structure and dynamics over the 45-year interval reported here. The moderate within-landscape environmental gradients sampled with the CARBONO Project experimental design had relatively small (soil nutrients) or non-detectable (slope) effects on the structure and dynamics of the forest plots.

It remains an open question why this intensively studied old-growth landscape fails to support the global-scale directional increases in biomass and demographic rates that have been reported for TRF [1]. As data accumulate over time, more relevant evidence should accumulate to evaluate directional trends in TRF. Even with increasing numbers of tropical-forest study plots, however, we do not think the B&FH_o_ is likely to be resolved in the near future. Instead, we believe that ultimately accepting or rejecting the B&FH_o_ will require a new paradigm in the way tropical forest science is carried out.

Most of the data to date that have been advanced to support the B&FH_o_ are not available for free and unconditional access. This means that independent review of each plot's siting and measurement protocols and of the raw data (including field data sheets) and subsequent analyses is impossible. A definitive acceptance or rejection of the B&FH_o_ would occur much more rapidly if scientists were able to independently analyze all published field studies. Tropical forest ecology has proceeded largely outside the general global movement towards open data access. As a result, the entire field is built on work that cannot be independently assessed. This is a fundamental weakness in tropical forest science, and is a glaring and startling anachronism compared to other scientific disciplines [48].

The Bigger and Faster Hypothesis is a critical paradigm regarding the status of the world's tropical forests, and the trends postulated by the hypothesis would have significant global impacts on carbon cycling, biogeochemistry and conservation of biodiversity [14]. Given the importance of this issue, it should be addressed through the tropical ecological research community establishing unrestricted access to the vast quantities of existing and future data as the norm. There is now no technical limitation to freely sharing all data and metadata, from digital databases down to scanned images of every field sheet. The only major limitation to universal unrestricted data access is researcher collaboration.

## Supporting information

S1 Fig Location of CARBONO project forest inventory plots. Locations of the 18 0.50 ha 50 x 100 m permanent forest inventory plots at the La Selva Biological Station, Puerto Viejo de Sarapiquí, Costa Rica. Plots were sited with a stratified random design within three principal upland landscape units: flat sites on old alluvial soils (plots shown in tan), flat ridgetops on more nutrient-residual soils (blue), and steep slopes on residual soils (red). Also shown are the two forest inventory plots (OTS Plot 1 and Plot 3) that were used for historical comparisons with forest structure and dynamics from the 1960's and the 1980's [16–18].
(TIF)

S2 Fig Stem density through time in three different edaphic conditions. Mean stem density in 18 0.50 ha plot in three edaphic conditions ±1 S.E.M.
(TIF)

S3 Fig Estimated basal area by edaphic condition. Mean basal area (±1 S.E.M.) in three different landscape types at the La Selva Biological Station, Costa Rica. N = 6 0.50 ha plots per edaphic category.
(TIF)

S4 Fig Recruitment. Mean rates of recruitment ± 1 S.E.M. for old growth forest in 18 0.50 ha plots at the La Selva Biological Station, Costa Rica.
(TIF)

S5 Fig Turnover. Mean rates of turnover ± 1 S.E.M. for old growth forest in 18 0.50 ha plots at the La Selva Biological Station, Costa Rica.
(TIF)

S6 Fig Multidecadal trends in recruitment. Multidecadal trends in recruitment in old-growth forests on residual and alluvial soils at the La Selva Biological Station, Costa Rica. Data from 1969–1982 are from OTS Plot 1 (4.4 ha, alluvial soil) and OTS Plot 3 (4.0 ha, residual soil) [17]). Data from 1997 onward are from the 6 0.5 ha CARBONO Project plots on alluvial soil and the 12 0.5 ha plots on residual soil (ridgetops and slopes).
(TIF)

S1 Table CARBONO plots static structure data. CARBONO plot stem inventory data 1997–2014 for 18 0.5 ha plots in old-growth Tropical Wet Forest at the La Selva Biological Station, Costa Rica.
(CSV)

S2 Table CARBONO plot growth data. CARBONO plot stem growth data 1997–2014 for 18 0.5 ha plots in old-growth Tropical Wet Forest at the La Selva Biological Station, Costa Rica.
(CSV)
