# Supplementary figures and images for: Multidecadal stability in tropical rain forest structure and dynamics across an old-growth landscape
Source: PLoS One. 2017 Oct 5;12(10):e0183819. doi: 10.1371/journal.pone.0183819 (PMC5628793; doi:10.1371/journal.pone.0183819)

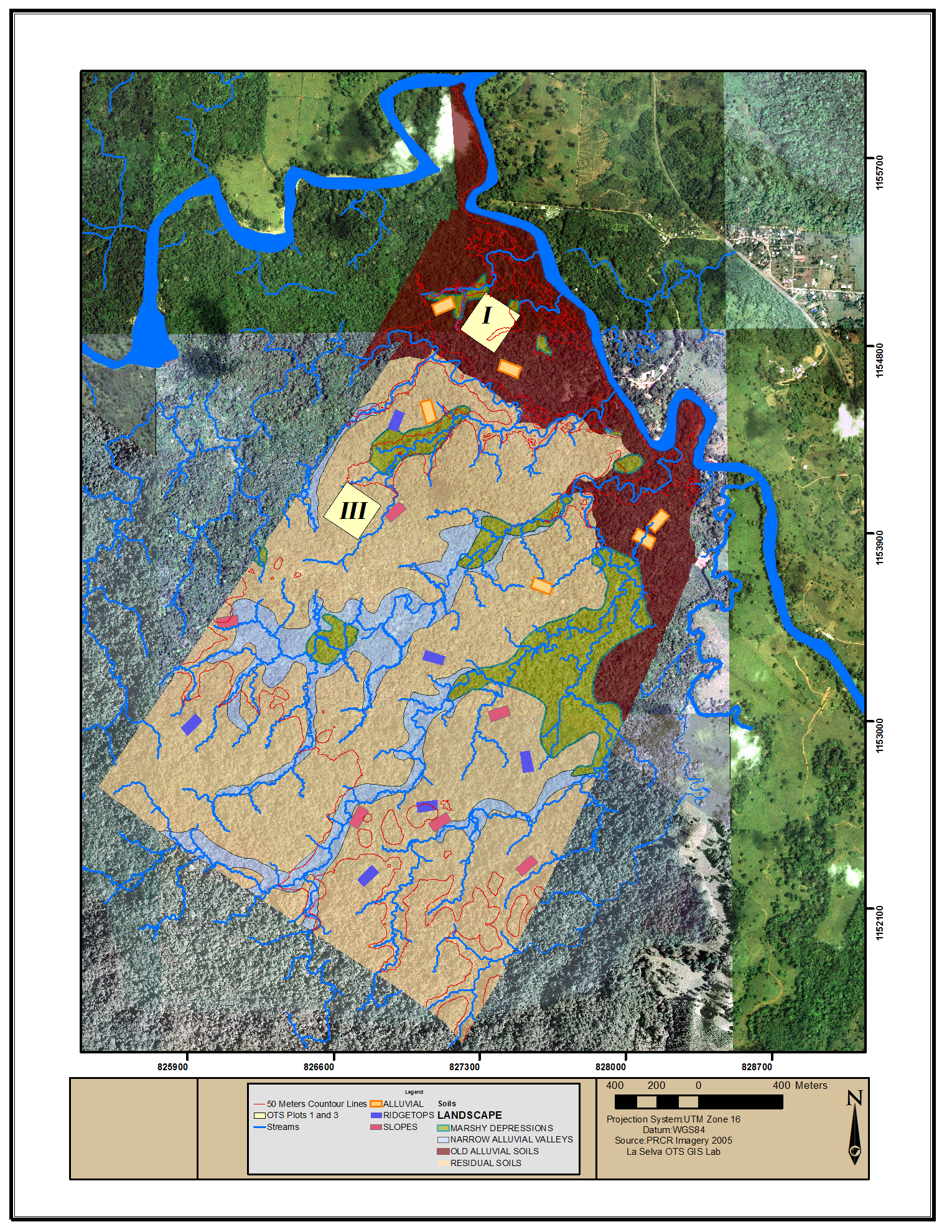

Supplement: S1 Fig — Locations of the 18 0.50 ha 50 x 100 m permanent forest inventory plots at the La Selva Biological Station, Puerto Viejo de Sarapiquí, Costa Rica. Plots were sited with a stratified random design within three principal upland landscape units: flat sites on old alluvial soils (plots shown in tan), flat ridgetops on more nutrient-residual soils (blue), and steep slopes on residual soils (red). Also shown are the two forest inventory plots (OTS Plot 1 and Plot 3) that were used for historical comparisons with forest structure and dynamics from the 1960's and the 1980's [16–18]. (TIF) [file pone.0183819.s001.tif]

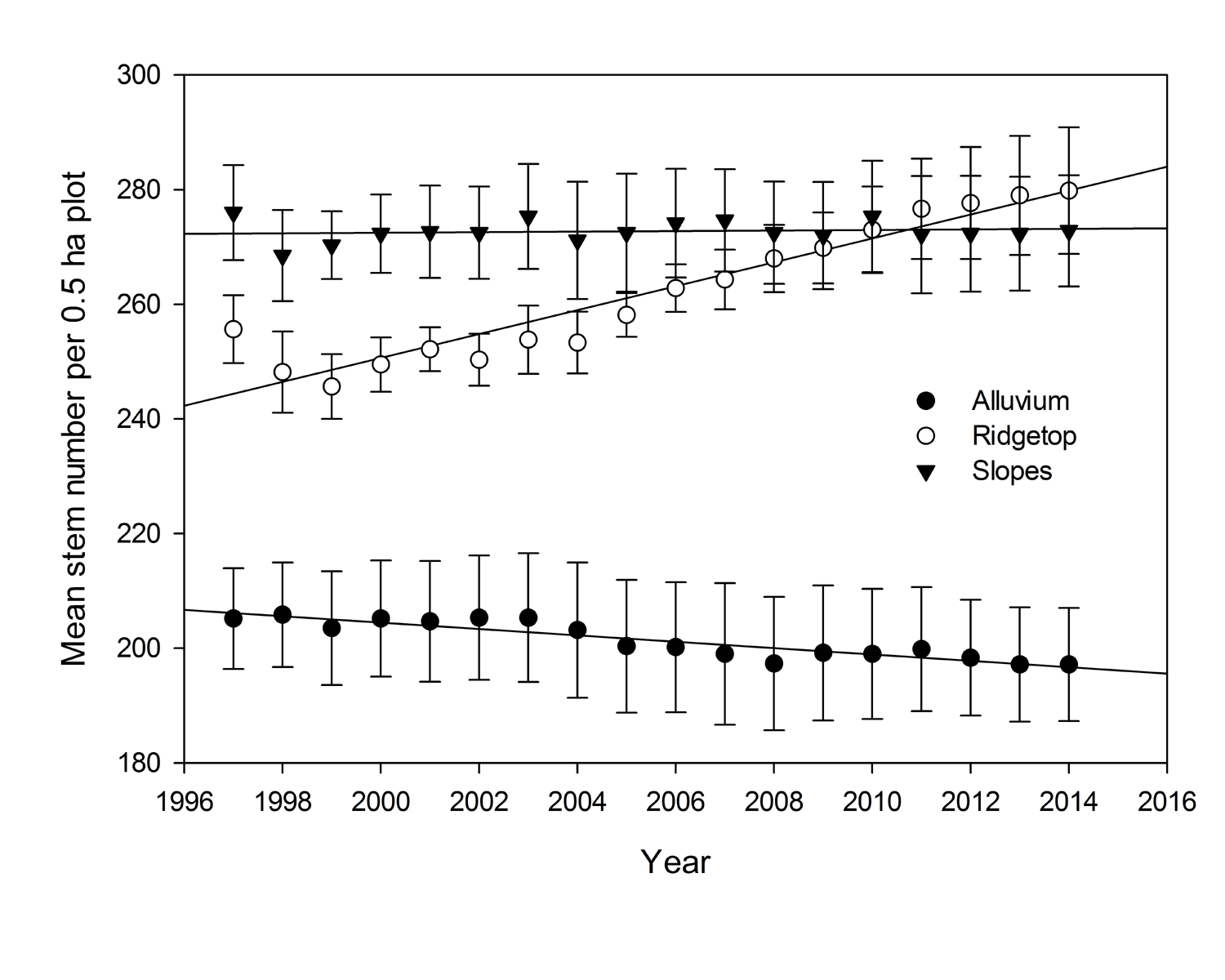

Supplement: S2 Fig — Mean stem density in 18 0.50 ha plot in three edaphic conditions ±1 S.E.M. (TIF) [file pone.0183819.s002.TIF]

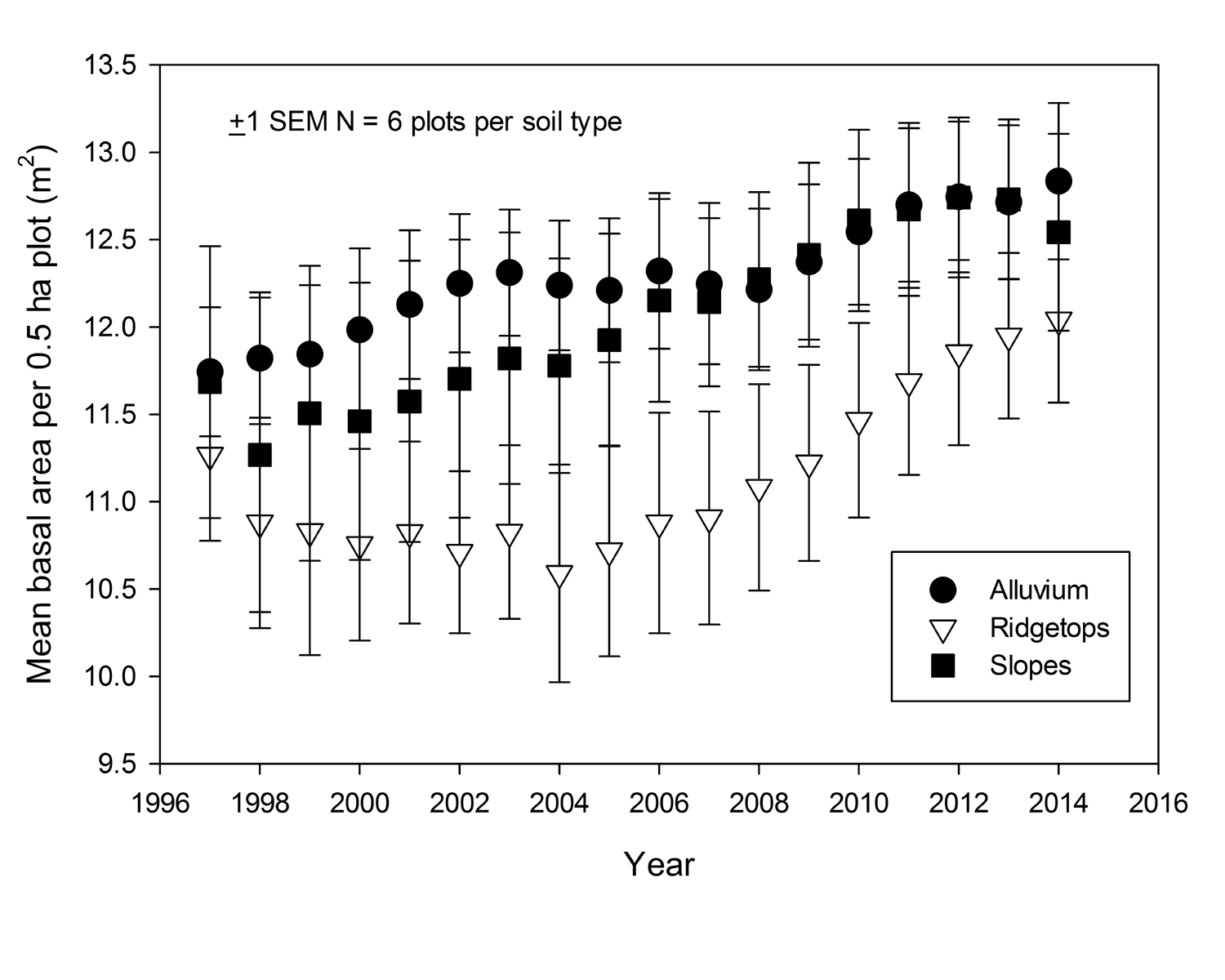

Supplement: S3 Fig — Mean basal area (±1 S.E.M.) in three different landscape types at the La Selva Biological Station, Costa Rica. N = 6 0.50 ha plots per edaphic category. (TIF) [file pone.0183819.s003.TIF]

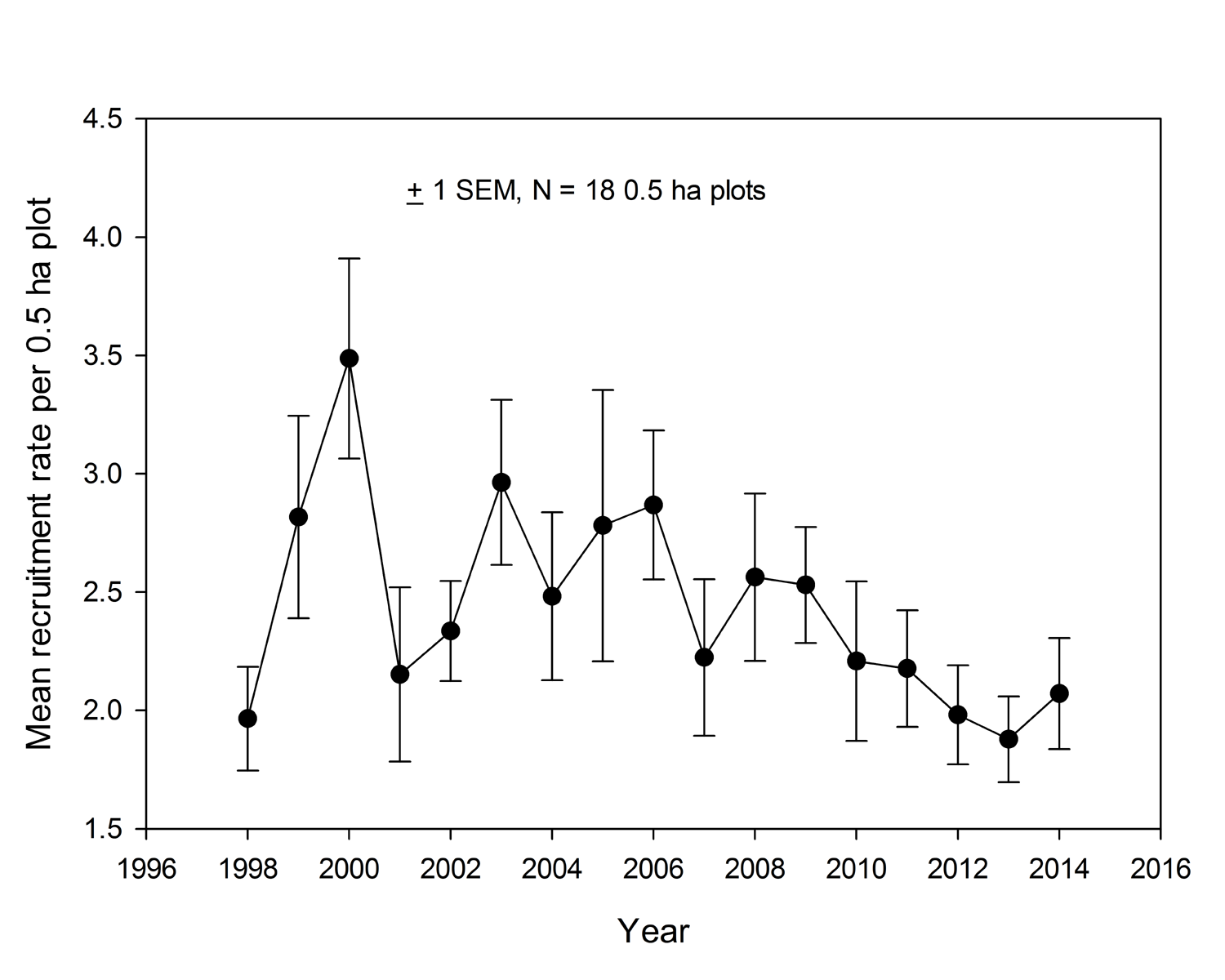

Supplement: S4 Fig — Mean rates of recruitment ± 1 S.E.M. for old growth forest in 18 0.50 ha plots at the La Selva Biological Station, Costa Rica. (TIF) [file pone.0183819.s004.TIF]

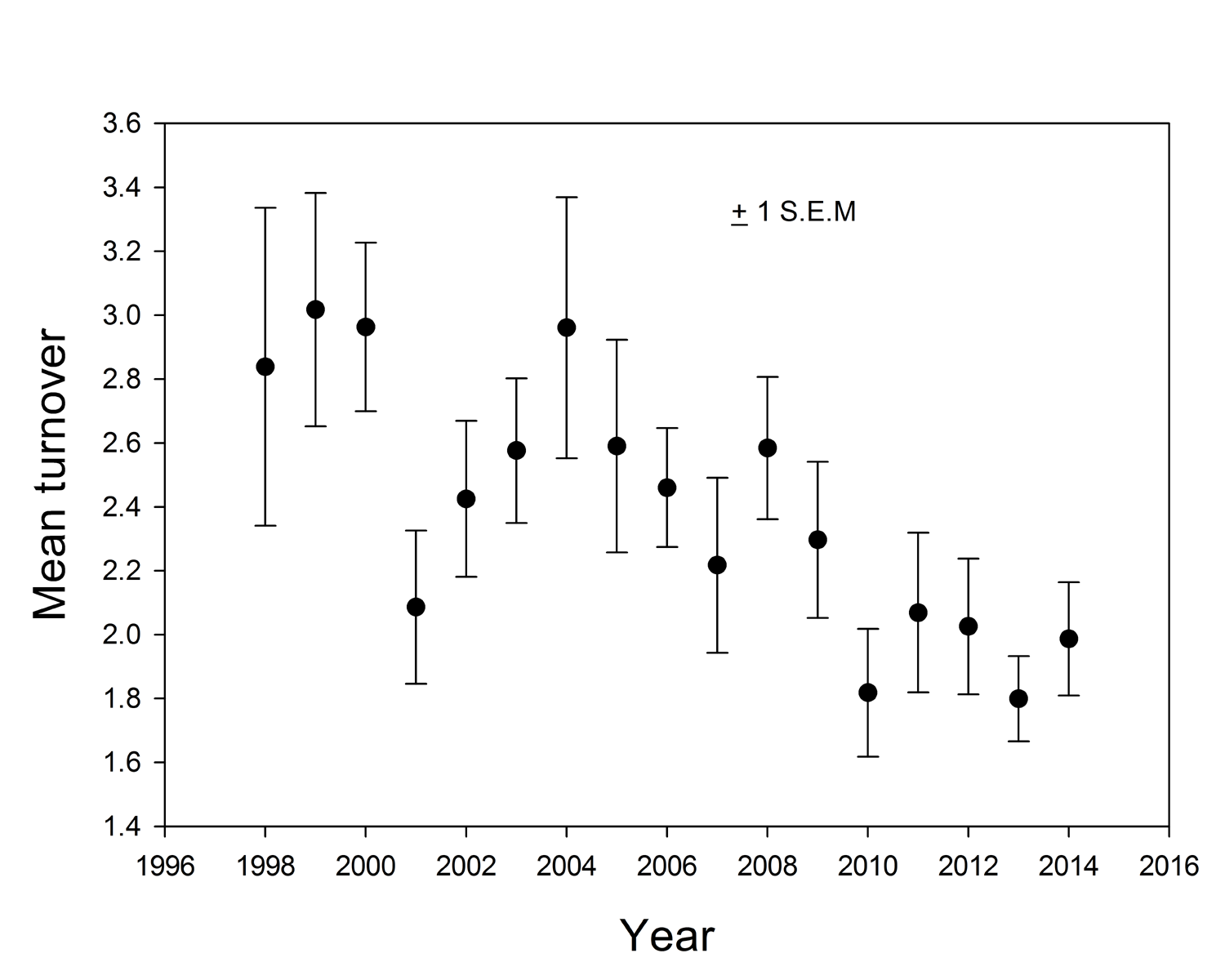

Supplement: S5 Fig — Mean rates of turnover ± 1 S.E.M. for old growth forest in 18 0.50 ha plots at the La Selva Biological Station, Costa Rica. (TIF) [file pone.0183819.s005.TIF]

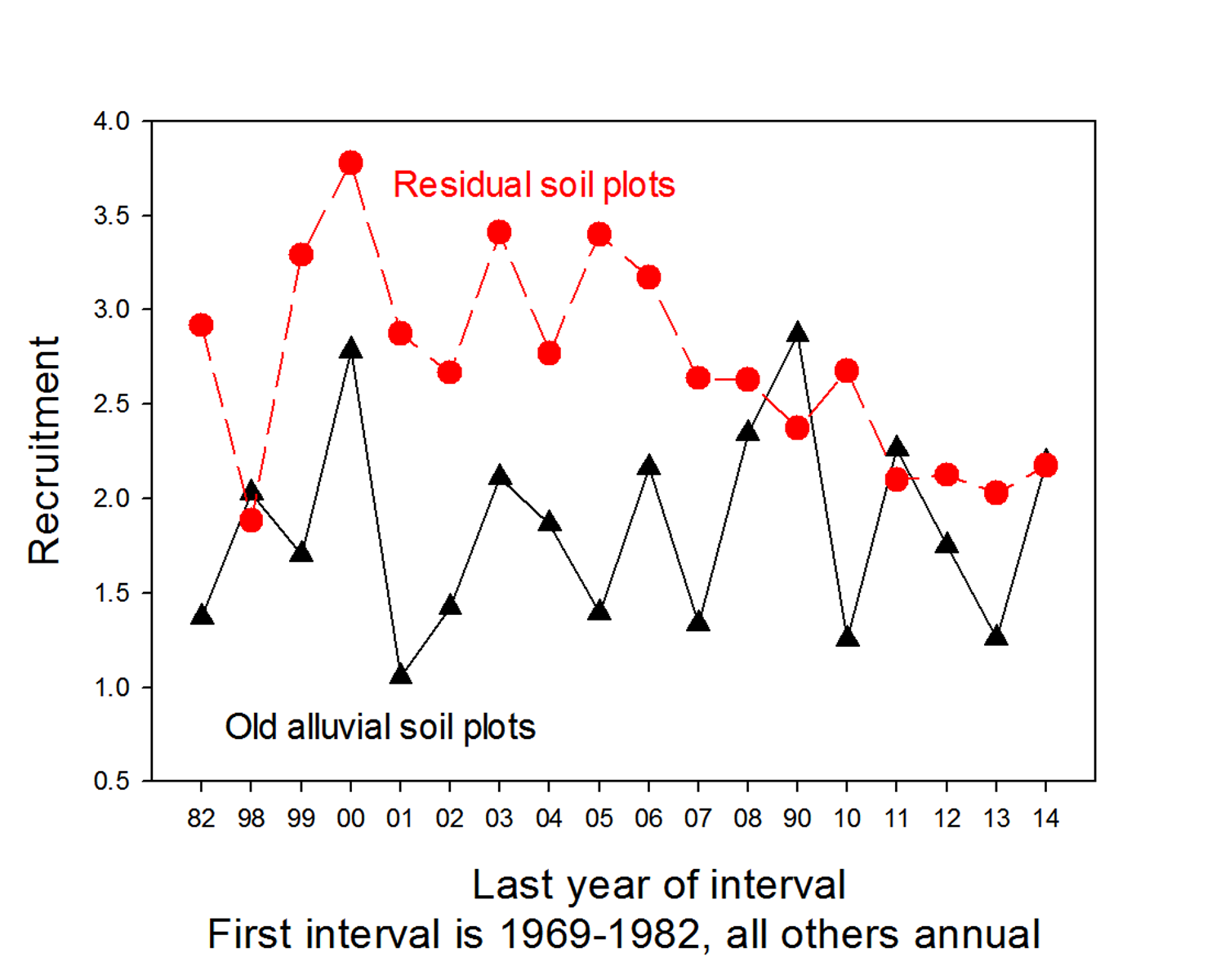

Supplement: S6 Fig — Multidecadal trends in recruitment in old-growth forests on residual and alluvial soils at the La Selva Biological Station, Costa Rica. Data from 1969–1982 are from OTS Plot 1 (4.4 ha, alluvial soil) and OTS Plot 3 (4.0 ha, residual soil) [17]). Data from 1997 onward are from the 6 0.5 ha CARBONO Project plots on alluvial soil and the 12 0.5 ha plots on residual soil (ridgetops and slopes). (TIF) [file pone.0183819.s006.tif]
